# Supplementary material for: A radioligand binding assay for the insulin-like growth factor 2 receptor
Source: PLoS One. 2020 Sep 2;15(9):e0238393. doi: 10.1371/journal.pone.0238393 (PMC7467306; doi:10.1371/journal.pone.0238393)
Supplement: S1 File — (PDF) [file pone.0238393.s001.pdf]

Supporting Information

S1 Fig. RP-HPLC chromatogram of the purified D11 protein.

S2 Fig. MALDI mass spectrum of purified D11 protein.

S3 Fig. RP-HPLC separation of crude products after radiolabeling of human IGF2 with [^{125}I].

S4 Fig. RP-HPLC analysis of isolated [^{125}I]-monoiodotyrosyl-Tyr2-IGF2.

S5 Fig. Representative binding curves of the hormones for insulin and IGF-1 receptors in membranes of intact cells.

S6 Fig. Relative abilities of human insulin, IGF1, IGF2, and Leu19-IGF2 to stimulate phosphorylation of receptors.

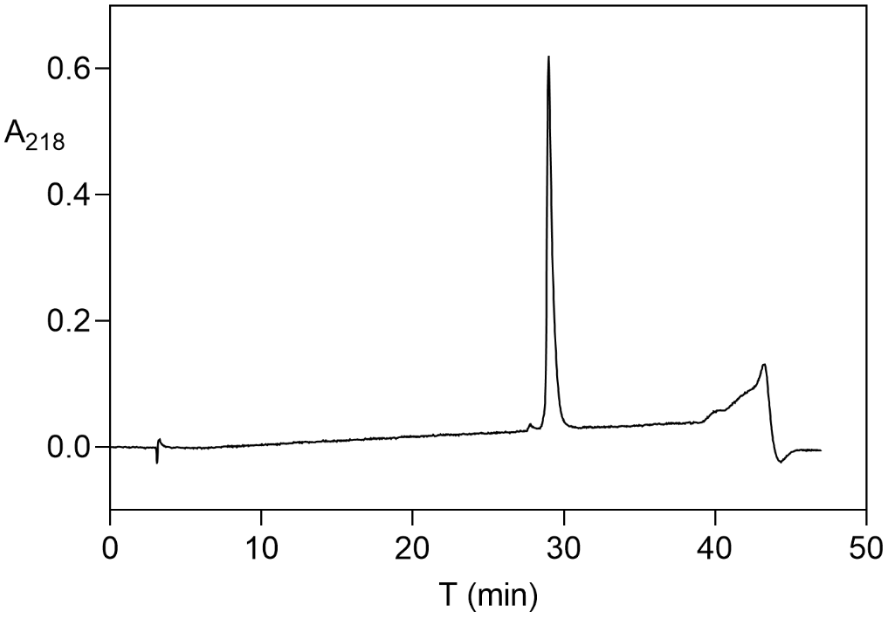

S1 Fig. RP-HPLC chromatogram of the purified D11 protein. Analysis was done on Vydac C4 column 0.4 x 25 cm in a gradient of acetonitrile in water with 0.1 % TFA.

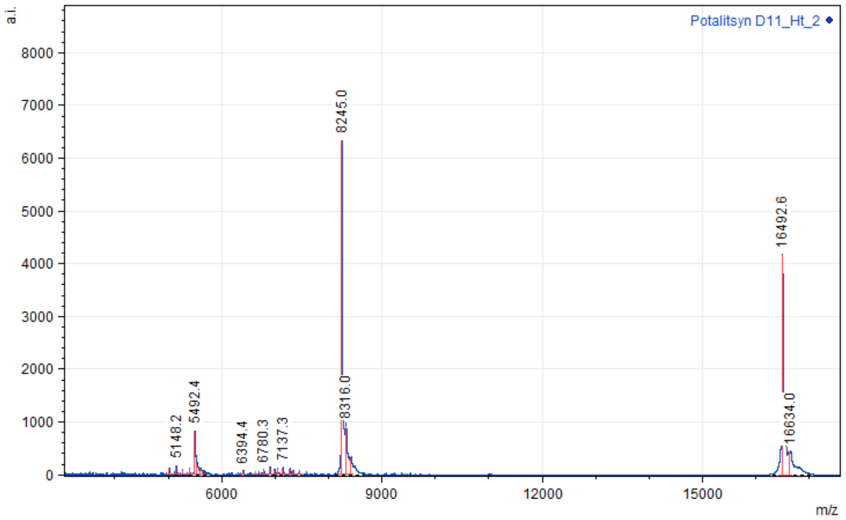

S2 Fig. MALDI mass spectrum of purified D11 protein. Expected average mass is 16491.6. The signal 8245.0 is a double charged molecule and the signal 16492.6 belongs to a single charged molecule.

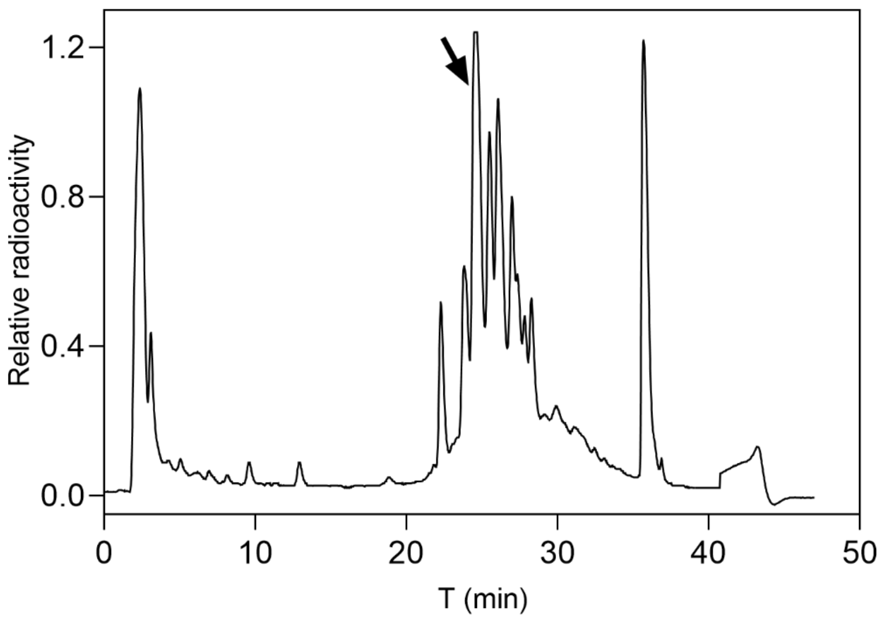

S3 Fig. RP-HPLC separation of crude products after radiolabeling of human IGF2 with [^{125}I]. The isolated peak containing [^{125}I]-monoiodotyrosyl-Tyr2-IGF2 is marked with an arrow. HPLC set up: BioZen C4 column (5μ, 125 × 4.6 mm), 1 mL/min. Solvent A = H_2O, TFA (0.1 %), solvent B = AcCN, TFA (0.1 %), gradient: 20 % B for 0 min., 54 % B in 80 min (linear).

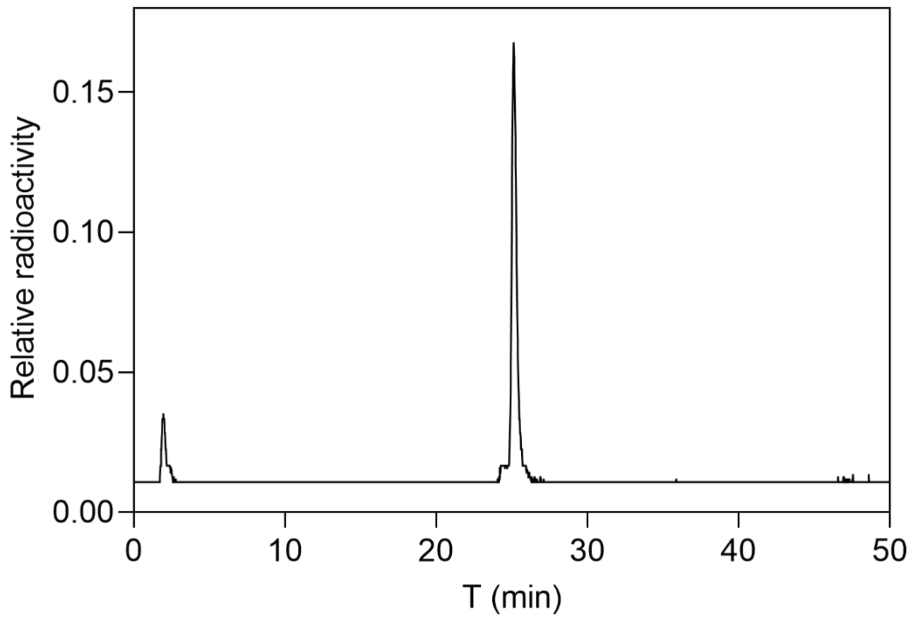

S4 Fig. RP-HPLC analysis of isolated [^{125}I]-monoiodotyrosyl-Tyr2-IGF2. HPLC set up was the same as in Figure S3.

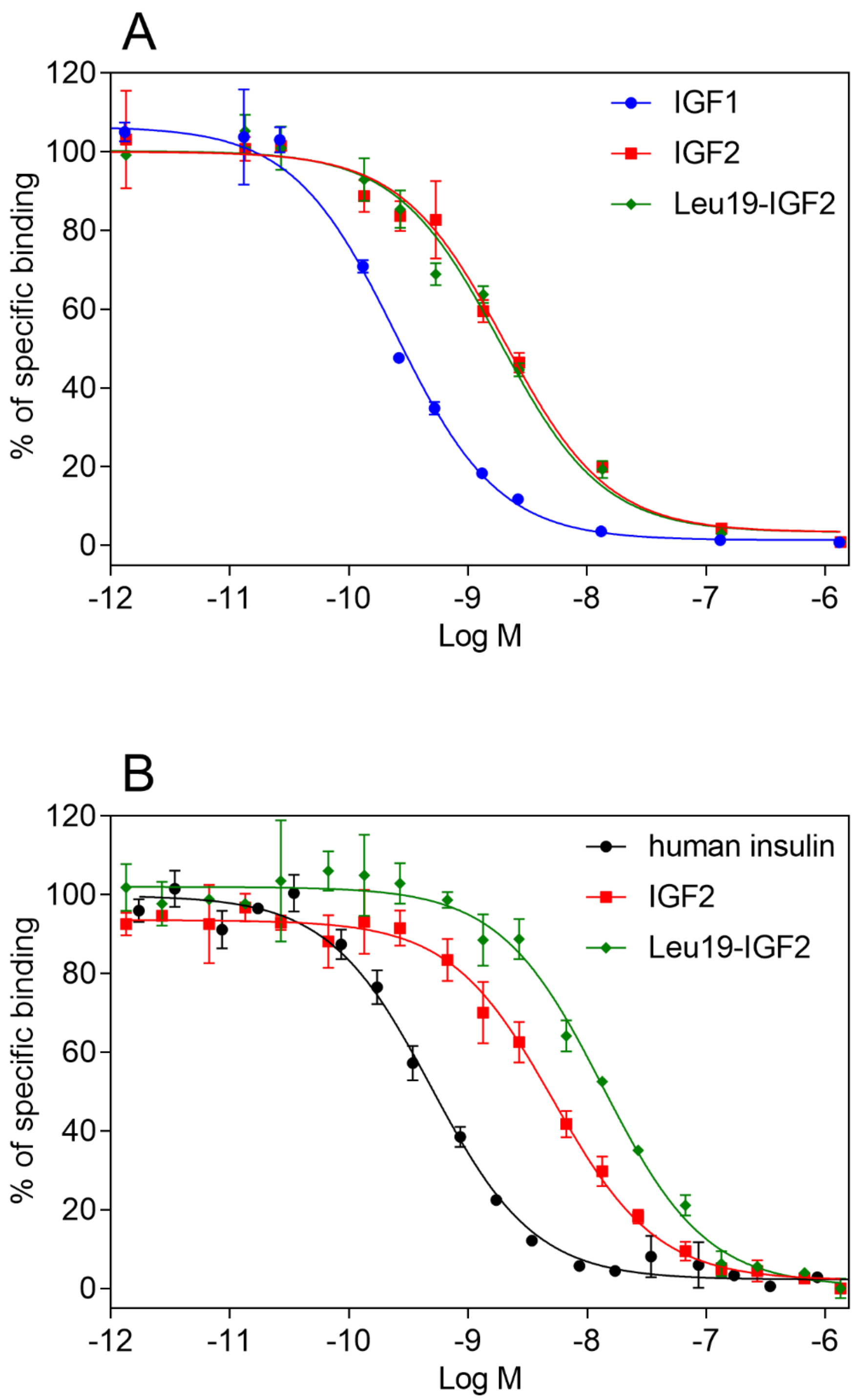

S5 Fig. Representative binding curves of the hormones for insulin and IGF-1 receptors in membranes of intact cells. (**A**) Inhibition of binding of human [^{125}I]-IGF1 to IGF1R in membranes of mouse fibroblasts by IGF1 (in blue), human IGF2 (in red), and Leu19-IGF2 (in green). (**B**) Inhibition of binding of [^{125}I]-monoiodotyrosyl-TyrA14-human insulin to IR-A in human IM-9 lymphocytes by human insulin (●), human IGF2 (■), and Leu19-IGF2 analog (♦).

A

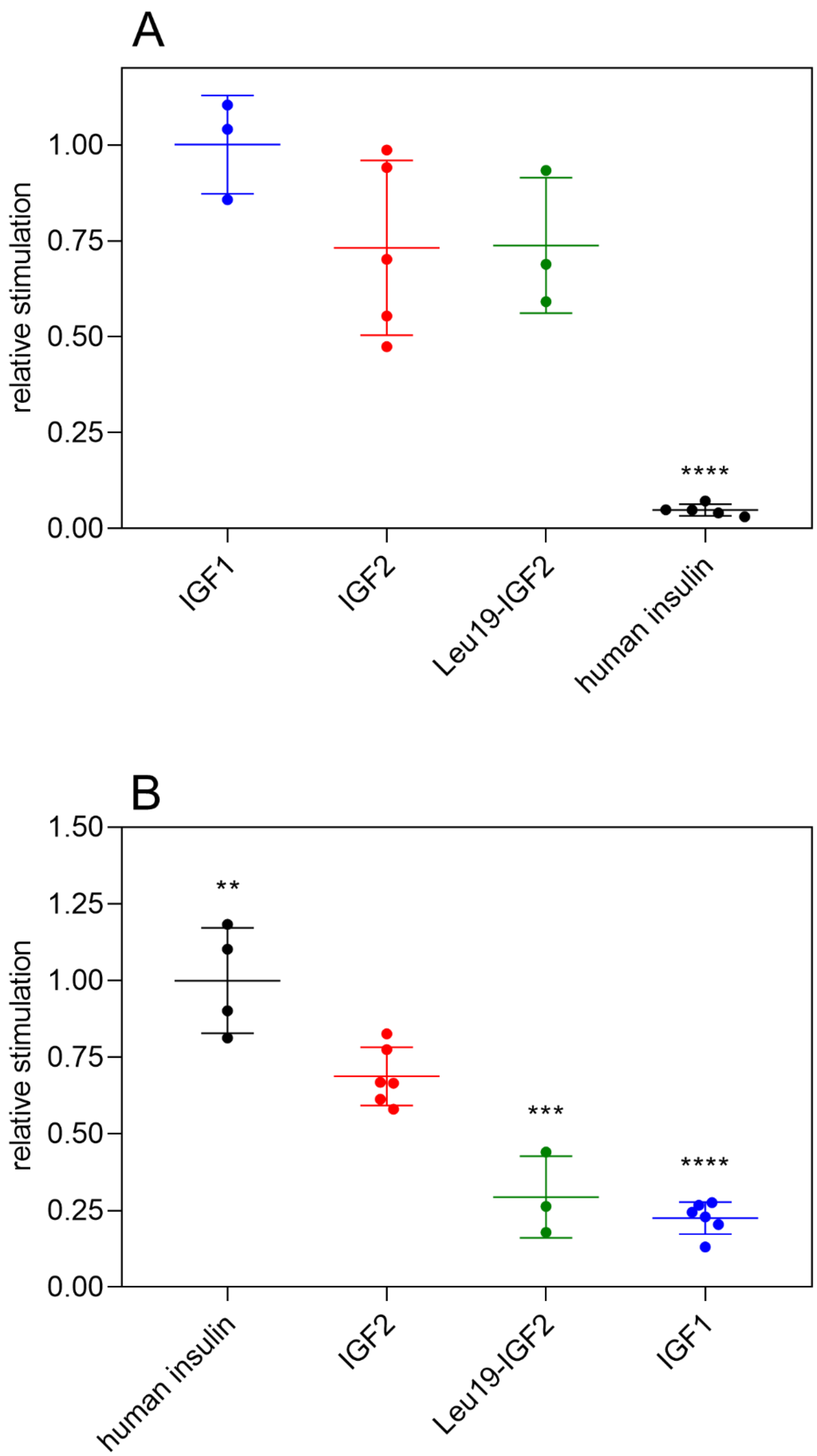

S6 Fig. Relative abilities of human insulin, IGF1, IGF2, and Leu19-IGF2 to stimulate phosphorylation of receptors. IGF1R-transfected cells (**A**) or IR-A-transfected cells (**B**) were stimulated with 10 nM ligands for 10 min. Asterisks indicate that phosphorylation of the

receptor induced by a ligand differs significantly from that of insulin (*, $p < 0.05$; **, $p < 0.01$; ***, $p < 0.001$, ****, $p < 0.0001$.
